# Medical News

**Published:** 1851-11

**Authors:** 


### MEDICAL NEWS.

THE TENNESSEE HOSPITAL FOR THE INSANE, situated about six miles from Nashville, is nearly completed. It is, we learn from the Nashville Journal of Medicine and Surgery, of the castellated style of architecture, 325 feet in length from east to west, and in breadth across the centre, 98 feet. It is said to be very beautifully situated, with two hundred and fifty-five acres of ground and farm land attached to it. Dr. JOHN S. YOUNG is the Superintendent.

Drs. J. C. WARREN, of Boston, and MÜTTER, of Philadelphia, who have been absent during the summer months in Europe, have returned to the United States.

Dr. J. V. C. Smith, the Editor of the Boston Medical and Surgical Journal, has, we are glad to see, returned from abroad, and resumed his editorial duties. Dr. Smith's interesting correspondenee with the 'Journal,' during his tour, was frequently noticed and quoted in the European medical journals.

Professor A. H. Buchanan, of the University of Nashville, returned from a European tour, in October last. Several pleasant and in teresting letters were communicated by him, during his absence, to the Nashville Journal of Medicine and Surgery.

South Carolina and Dr. Clark. Complimentary to the Profession.—To prove that medical men do not always go unrewarded by those to whom important services may have been rendered, we give the following instance as an exception. The State of South Carolina has recently presented to her distinguished son, Dr. C. J. Clark, a gold medal, weighing two and a half oz., upon one side of which is a representation of the landing of Vera Cruz; and on the other, the South Carolina "Coat of Arms." This honor has been conferred in consideration of the valuable services rendered by Dr. Clark to her brave sons who fought and many of whom fell, upon the bloody plains of Mexico, in maintaining the honor of the State and in defending our common country. South Carolina knows how to encourage talent, and to reward true merit —and in this she has given us many noble examples worthy of her distinguished name.

We hope Dr. Clark will not feel envious when he hears in what manner we were rewarded for three months' services on the Rio Bravo, Mexico. Ours came from head-quarters—Washington city. In turning over to the Quarter-Master at this place the medicine and hospital stores in our charge, one or two silver-washed catheters were missing; these were promptly reported to head-quarters, and in due time a bill of $40 was returned to us, claiming indemnity for that which was loaned and used up in the service of the country.

From that day to this our thirst for military glory has been on the wane, and we wish henceforward to be regarded as an uncompromising advocate for the "Peace Congress."—*New Orleans Med. and Surg. Journal.*

Dr. Bennett Dowler.—Dr. Dowler, of New Orleans, author of numerous scientific papers containing original and ingenious experimental observations on various physiological subjects, has been appointed to the Chair of Physiology and Pathological Anatomy, in the Memphis (Tenn.) Medical College. This institution has lately been reorganized, and a lecture-session is announced for the coming winter.

DEATH OF SYLVESTER GRAHAM.—Most of our readers in this part of the country are familiar with the name of this individual, who some years since made himself notorious in urging upon the community the system of light and exclusively vegetable diet to which his name was given. He died lately at Northampton, in Massachusetts, at the age of about 50. The Gazette, of that place, states that his health had been gradually failing for the last year, and he had suffered much from rheumatism in his hands and feet. "Post-mortem examination disclosed no disease in the system, which, in the opinion of the medical examiners, was sufficient to produce his death; and the immediate cause of his decease is thought to be the use, contrary to the advice of his physician and friends, in the extreme exhaustion of the system, of Congress water and a tepid bath."

PROSECUTION FOR MAL-PRACTICE.—A case has recently been tried in the Court of Common Pleas, in the city of Lowell, of a character similar to those mischievous prosecutions that for some years past have alarmed medical gentlemen, in various parts of the country, to such a degree that many have about concluded to let all surgical patients go unassisted in their afflictions. If by any combination of unforeseen constitutional or other circumstances, a fractured bone or a punctured wound is not immediately cured, the surgeon is at once driven to the wall by a prosecution for mal-practice, the object and aim of the prosecutors being to make to themselves mammon out of the spoils, even if they rob the practitioner of his honest earnings and ruin his reputation. It seems that, in the case alluded to, a woman cut her right thumb while paring apples. In consequence of the condition of the wound, which pained her, Dr. J. T. G. Leach, of that city, was called in for advice. From a careful analysis of the testimony in regard to the treatment, his course appears to have been perfectly judicious, and this was the opinion of very eminent medical gentlemen who were called before the court. Notwithstanding the weight of evidence would seem to have been strong enough to have sustained Dr. Leach, to our surprise, at least, the jury could not agree. The more we have reflected upon the testimony, the more we are astonished at the result. Of what value is evidence in courts of law, if it is to have no weight? Dr. Charles A. Savory, Dr. D. Mowe,

Dr. Thurston, Dr. J. C. Dalton, Dr. Green, Dr. Parkman, and Dr. Balch, if we understand them, sustained Dr. Leech triumphantly.

At the Missionary Hospital, in China, in which Dr. Peter Parker, the American Surgeon, has distinguished himself by the performance of some of the boldest operations in surgery, before the people became enlightened and comprehended the value of his services he refused to operate till a bond had been executed, in which applicants agreed not to claim damages of him, should he be unsuccessful. It would perhaps be the course of prudence for surgeons among us to keep blank bonds on hand, to be filled up before commencing treatment, if the public continue to ask for their best endeavors and then prosecute them for damages should the result be unsuccessful.—*Boston Med. and Surg. Journal.*

OBITUARY.—Dr. WHEATON, of Providence, R. I., died lately in that city, at the patriarchal age of 90 years. "He was distinguished during his long life, for the virtues of a Christian, the acquirements of a gentleman, and the acquisitions of a scholar."

Dr. REYER, chief physician to the Royal Hospital at Vienna, lately committed suicide by the use of chloroform.

Dr. HENRY K. BURROUGHS died at Savannah, on the 13th of October. He had been Mayor of the city, and was highly esteemed.

Dr. ALPHEUS F. STONE, of Greenfield, Massachusetts, President of the Franklin County Medical Society, of that State, died on the 5th of September last, in the 74th year of his age. Dr. Stone had been in extensive practice for fifty years, and was greatly esteemed in his native State.

Dr. OKEN.—We have just received intelligence of the death of the famous naturalist, Dr. Lorenzo Oken, whose theory of the Cranial Homologies effected a revolution in philosophical anatomy, and led the way to the admirable researches of our own Owen. The name of Oken is, in this country, most commonly associated with his "Physico-philosophy," a translation of which work, by Mr. Tulk, was published by the Ray Society. It abounds in admirable generalization, unfortunately immersed in much that is false and fantastic, and clothed in the cloudiest phraseology of German transcendental metaphysics. Oken's researches and speculations (for he was as practical as he was dreamy) extended over all departments of natural history. Of the value he set upon facts, and the industry with which he collected them, a lasting monument exists in the volumes of the "Isis," a vast library of abstracts of the science of his time, founded and conducted by him as a periodical. Few men have had greater influence on European science than Oken. Until forced to quit Germany on account of his political opinions, he held a Professorship at Jena. Latterly, he was Professor of Natural History at the University of Zurich, in which city he died a few days since, at the advanced age of seventy-three years.—*Literary Gazette.*

THE LATE DR. KIDD.—By the death of Dr. Kidd, formerly professor of Chemistry, and since 1822 Regius Professor of Medicine, the University of Oxford has just lost one of the most active of its men of science, who belonged, by their chief works and labors, to a former generation. Dr. Kidd did good service in his time, as his publications testify, in various departments of mineralogical, chemical, and geological research, and about ten years ago, his last appearance, we believe, as an author, he put forth some observations on medical reform. Dr. Kidd was one of the eminent men selected under the Earl of Bridgewater's will to write one of the well known "Bridgewater Treatises," and we believe it has gone through more editions than any work of the series. The subject was, "On the Adaptation of External Nature to the Physical Condition of Man." Together with the Regius Professorship of Medicine, to which the mastership of Ewelme Hospital, in the county of Oxford, is attached, Dr. Kidd held the office of librarian to the Radcliffe Library.

DEATH OF DR. BAUDELOCQUE.—This distinguished physician has lately sunk under a chronic malady, aged 55. He is well known to English readers as a writer on obstetrical subjects, and on scrofula. He was physician to the Hospital for sick children, and had one of the largest midwifery practices in Paris.

COFFINISM.—Under the name of Coffinism, Thompsonism appears to be making some head-way in Great Britain. We find the following allusions to our old acquaintance, under its new name, in the *London Pharmaceutical Journal*, for September:

"Coffinism is another variety of quackery, addressed to a different class of persons, from homœopathy. There is no disguise or mummery in this system. It is an open and impudent repudiation of medical science, physiology, and anatomy. The entire code of practice is comprised in Dr. Coffin's book; the practitioners are shoe-makers, chandlers, mechanics, &c. The remedies are medicinal plants, some of which are powerful poisons. They are not administered in infinitesimal doses, but by the tea-spoonful or pennyworth; and for the protection of the practitioners when inquests occur, it has been found expedient to establish a 'Defence Fund," to which all subscribe, and in the benefit of which each person participates when in trouble. During the past month a "Medico-botanic Demonstration" was made, occupying four days, during which time the votaries visited Dr. Coffin's Museum, Catlin's Museum, and Kew Gardens; they drank tea at Tabernacle Row, Finsbury; they assembled in Trafalgar Square, and proceeded "in procession through Pall Mall to the Crystal Palace, with appropriate banners, and headed by Drs. Coffin and Harle." The profits of the entertainment go to the "Defence Fund." This description of quackery is quite as formidable as the "Do-nothing" system. It is practiced among a class of persons who are less able to take care of themselves than the wealthy dabblers in globules, and it is gaining ground to an extent which must ultimately effect its own cure.

A mother thinks her child looks dull, she takes him to a Coffinite cobbler, who administers a tea-spoonful of lobelia inflata; the child becomes worse, the dose is repeated. The child dies, an inquest is held. Dr. Coffin comes forward with his "defence fund," the jury are made to believe that the death of the child may be attributed to the treatment of the medical man who was called in at the last extremity. The cobbler is acquitted, and returns to his practice, impressed with the conviction that if the child had taken another dose of his medicine the catastrophe would not have occurred."

Munificent Legacy.—Dr. Jecker, who has left various useful works on physiology and microscopal anatomy, has just died, and, by his will, gives the Academy of Sciences at Paris the sum of £8000, to found an annual premium, to be awarded to the author of the most useful work on organic chemistry.

Medical Periodicals in Egypt.—We have received the numbers for June and July of a very interesting monthly periodical, published at Alexandria, whose title is *Giornale Optamologico Egiziano.* This paper is written in Italian, and edited by Dr. Abbate; the size is but half a sheet, but the articles, both original and copied, are well selected, and there is no doubt but this journal might be made very useful, emanating, as it does, from a country where ophthalmic affections are unfortunately so frequent. In the June number there are some good remarks on staphyloma, myopia, and presbyopia, &c.; the former are continued in the number for July, with interesting articles on the good effects of valerianate of zinc and hyoscyamus in rheumatic ophthalmia, on the hygienic means to be used in Egypt to avoid affections of the eye, &c., &c.—*London Lancet.*

Was the Roman Army provided with any Medical Officers.—In a late pamphlet under this title Dr. Simpson describes a tablet discovered at Housesteads, in Northumberland (the ancient Borcovicus) one of the principal stations on the great defensive wall, reared in the second century by the Emperor Hadrian. From this monument, which was raised by his companions "to Anicius Ingenuus, Physician in Ordinary of Cohort the first of the Tungrians," we learn that more than one medical officer was attached to each cohort. Further evidence is also adduced by Dr. Simpson, rendering it probable that there were superior medical officers placed over the staff of the legions.

Study of Anatomy in Egypt.—At the time when Mahomet Ali was persuaded by Clot Bey to organize a school of medicine in Cairo, the old man consulted the council of the Ulama, or learned men of the orthodox sects, in regard to the legality of dissecting human bodies, for the purpose of anatomical knowledge. They decided that it would be repugnant to religion. However, he said nothing, and gave directions to his surgeon to proceed. He had the united force of Musselman prejudices and Moslem bigotry marshalled against him; but he was the pillar that sustained the entire fabric of Egyptian government, against whose orders no one dared raise the voice of remonstrance, however much they might growl over an infraction of the Koran. He lived long enough to have his bigoted subjects become familiar with dissections, and till the four orthodox sects of the Hanafees, Shafees, Màlikees and Hambelees, had lost their influence over the ignorant rabble of the metropolis. Anatomical pursuits are now conducted there on a proper scale, and under fewer restrictions than in several of these United States, where a physician and surgeon are required to understand anatomy in order to practice, and yet they are liable to prosecution, to fine and imprisonment, for doing precisely what they are required to do. Mahomet Ali was in advance of the age in his own dominions, and far wiser and more liberal than some of the enlightened christian legislators of these United States.—*Boston Med. and Surg. Journal.*

